# Novel *MYO1D* Missense Variant Identified Through Whole Exome Sequencing and Computational Biology Analysis Expands the Spectrum of Causal Genes of Laterality Defects

**DOI:** 10.3389/fmed.2021.724826

**Published:** 2021-09-13

**Authors:** Rabab Said Alsafwani, Khalidah K. Nasser, Thoraia Shinawi, Babajan Banaganapalli, Hanan Abdelhalim ElSokary, Zhaher F. Zaher, Noor Ahmad Shaik, Gaser Abdelmohsen, Jumana Yousuf Al-Aama, Adam J. Shapiro, Osman O. Al-Radi, Ramu Elango, Turki Alahmadi

**Affiliations:** ^1^Department of Medical Laboratory Technology, Faculty of Applied Medical Sciences, King Abdulaziz University, Jeddah, Saudi Arabia; ^2^Princess Al-Jawhara Center of Excellence in Research of Hereditary Disorders, King Abdulaziz University, Jeddah, Saudi Arabia; ^3^Department of Genetic Medicine, Faculty of Medicine, King Abdulaziz University, Jeddah, Saudi Arabia; ^4^Department of Pediatrics, Faculty of Medicine, King Abdulaziz University, Jeddah, Saudi Arabia; ^5^Pediatric Cardiac Center of Excellence, King Abdulaziz University Hospital, King Abdulaziz University, Jeddah, Saudi Arabia; ^6^Department of Genetics, Al Borg Medical Laboratories, Jeddah, Saudi Arabia; ^7^Pediatric Cardiology Division, Department of Pediatrics, Cairo University, Kasr Al Ainy Faculty of Medicine, Cairo, Egypt; ^8^Division of Pediatric Respiratory Medicine, McGill University Health Centre Research Institute, Montreal Children's Hospital, Montreal, QC, Canada; ^9^Department of Surgery Faculty of Medicine, King Abdulaziz University, Jeddah, Saudi Arabia; ^10^Pediatric Department, Faculty of Medicine in Rabigh, King Abdulaziz University, Jeddah, Saudi Arabia

**Keywords:** laterality defects, whole exome sequencing, microfilament, gene expression, variant

## Abstract

Laterality defects (LDs) or asymmetrically positioned organs are a group of rare developmental disorders caused by environmental and/or genetic factors. However, the exact molecular pathophysiology of LD is not yet fully characterised. In this context, studying Arab population presents an ideal opportunity to discover the novel molecular basis of diseases owing to the high rate of consanguinity and genetic disorders. Therefore, in the present study, we studied the molecular basis of LD in Arab patients, using next-generation sequencing method. We discovered an extremely rare novel missense variant in *MYO1D* gene (Pro765Ser) presenting with visceral heterotaxy and left isomerism with polysplenia syndrome. The proband in this index family has inherited this homozygous variant from her heterozygous parents following the autosomal recessive pattern. This is the first report to show *MYO1D* genetic variant causing left–right axis defects in humans, besides previous known evidence from zebrafish, frog and *Drosophila* models. Moreover, our multilevel bioinformatics-based structural (protein variant structural modelling, divergence, and stability) analysis has suggested that Ser765 causes minor structural drifts and stability changes, potentially affecting the biophysical and functional properties of *MYO1D* protein like calmodulin binding and microfilament motor activities. Functional bioinformatics analysis has shown that *MYO1D* is ubiquitously expressed across several human tissues and is reported to induce severe phenotypes in knockout mouse models. In conclusion, our findings show the expanded genetic spectrum of LD, which could potentially pave way for the novel drug target identification and development of personalised medicine for high-risk families.

## Introduction

Laterality defects (LDs) are a group of developmental diseases that affect internal organ positioning in the body. In general, human LDs can be divided into three categories: (1) *situs solitus* (SS) with normally expected organ arrangement; (2) *situs inversus* (SI) characterised by complete mirror image of organs; and (3) *situs ambiguus* (SA) with organ arrangement falling along a spectrum of various anomalies between SS and SI, including congenital heart defects (CHDs). Within SA, a subgroup of patients presents a severe and complex form of congenital heart disease, which is commonly known as *heterotaxy* (1). Defective left–right (LR) patterning of internal organs is associated with multiple congenital diseases affecting the cardiovascular system, kidneys, liver, and biliary tract (2, 3). According to the National Birth Defects Prevention Study (4), the estimated prevalence of LD is 1.1 per 10,000 in the United States. Despite the rare likelihood of LD, its incidence is excepted to be higher among the Arab population due to their high rate of consanguinity and genetic disorders (5).

The aetiology of LD is complex and includes both environment (5–7) and genetic factors (8, 9). Disease-causing genetic variations are found in <20% of LD cases; and the remaining 80% of cases are due to unidentifiable causes (10, 11). Up to now, known LD genes were mostly associated with NODAL/TGFβ signalling (*NODAL, CFC1, ACVR2B, LEFTYB, GDF1, TGFBR2*, and *FOXH1*), SHH signalling (*ZIC3* and *LZTFL1*), and monocilia function (*NPHP2, NPHP3, NPHP4, PKD2*, and *TTC8*) (10). Other genetic alterations associated with early cardiac development (*NKX2-5, CRELD1, MMP2*1, and *PKD1L1*) were also implicated in LD development (10). The main functional roles of these genes were demonstrated in LR axis determination, controlling cardiac looping direction, nodal activity regulation in embryogenesis, protein interaction of primary cilia, and signalling involved in morphogenesis cascade (12–19). Hence, LDs occur in a variety of different diseases, affecting various cardiac, respiratory, and gastrointestinal organs, reflecting the complex genes involved in signalling pathways of organogenesis and ciliary function.

Genetic testing and molecular diagnostics are now regarded as an useful approach to discover molecular causes underlying the LD development. Whole-exome sequencing (WES) analysis proved to be a successful method to uncover novel candidate genes or novel variants in known candidate genes (10). To this end, there is an increasing need to study the rare developmental disorders across different ethnic populations due to its potential in expanding the genetic spectrum of the disease. However, literature searches reveal sparse data on the Arab LD patients. We hypothesise that genetically investigating LD patients from a consanguineous Arab society will offer new insights into disease pathogenesis by identifying novel genes or novel variants in known genes, as demonstrated in other complex diseases (20). Therefore, the objective of the present study was to identify the genetic cause of LD in Arab patients, using WES and multilevel bioinformatics-based structural (protein variant structural modelling, divergence, and stability) and functional (gene expression and knockout mouse model) analysis approaches.

## Materials and Methods

The ethical approval for the present study was obtained from the institutional ethics committee of King Abdulaziz University Hospital (KAUH), Jeddah, Saudi Arabia. Informed consent forms were collected from both adult parents and their children (parental consent and children's assent for those <18 years old) prior to blood sample collection and genetic testing. As per the National Birth Defects Prevention Study, LD participants were selected based on the following clinical criteria: *situs inversus*, CHDs (heterotaxy), isomerism of the lungs (bilateral two lobes/left-sidedness and bilateral three lobes/right-sidedness), abdominal situs abnormality (*abdominal situs inversus* and SA), and spleen abnormality (asplenia and polysplenia) (4). One LD index family composed of the proband (affected child) and both parents was recruited to paediatric cardiology, surgery, and pulmonology clinics in KAUH. Clinical, laboratory, and radiological results were independently assessed by both paediatric cardiology and pulmonology consultants. Family pedigree was drawn by interviewing the parents. Samples from two other LD families were screened for the presence of the identified candidate variants.

### Molecular Testing

#### Clinical Sampling

A total of 5 ml of whole EDTA peripheral blood samples was collected from each study participant.

#### DNA Extraction

The genomic DNA was isolated from circulating lymphocytes using QIAamp DNA blood Kit as per the manufacturer's protocol and quantified using a NanoDrop 2000 spectrophotometer. DNA integrity was checked on 2% agarose gel electrophoresis.

#### Whole-Exome Sequencing Analysis

The DNA library was prepared using Agilent Sure Select Target Enrichment Kit. DNA library was captured using ultralong 120 mer biotinylated cRNA baits. The library was sequenced using HiSeq2000 Next Generation Sequencer (Illumina, San Diego, CA, USA). The FASTQ format sequence was obtained, and reads were aligned using Burrows-Wheeler Aligner (BWA) software (Version bwa-0.7.12) against human genome reference sequence build 38 (GRCH38.p12). Variant calling was conducted using the genome analysis tool kit (GATK). The filtration pipeline was applied as follows: all coding variants that passed quality control (phred > 30 score) were included. All variants with a minor allele frequency (MAF) <0.015% were included. Known candidate gene variants were filtered based on the function of the gene and their role in LD development. Genes that are related to LD disease were collected through the Coremine Medical™ tool, National Center for Biotechnology Information (NCBI), OMIM, and literature review.

#### Variant Validation Using Sanger Sequencing Method

The potential LD candidate variant was validated using Sanger sequencer ABI 3500 Genetic Analyzer. The primers were designed using NCBI Primer Blast to capture targeted mutation in candidate gene. The sequence files (chromatogram) were analysed using BioEdit software.

### Functional Analysis of Laterality Defect Variant Using Computational Methods

#### Amino Acid Conserved Domains

The functional relevance of LD candidate genetic variant on candidate proteins was predicted by searching the nucleotide and amino acid sequences against the functional domains of concerned protein as per the listing available in Conserved Domain Database (CDD). To estimate the sequence conservation characteristics of the functional domains in the candidate protein, CDD tool uses RPS-BLAST, which rapidly scans the query protein for pre-computed position-specific scoring matrices (PSSMs). The output file demonstrates the links between protein domains with annotations against the query input sequence together with imagining choices (21).

#### 3D Protein Modelling

The pathogenic effects of amino acid variants on disease candidate proteins can be best understood when they are studied at the structural level. Therefore, the potential effect of LD variant on the tertiary structural features was explored through 3D simulation of the candidate protein. Based on the availability of the X-ray crystallographic structure of the query protein, either a combination of *ab initio* approaches or homology modelling approaches were followed (22, 23). The 3D simulated structure of the native protein was then used to construct the mutated version of candidate protein, which was then energy minimised and then analysed for structural deformities like amino acid or whole structure level deviations using YASARA software (24). The impact of candidate variant on the stability of protein structure was estimated using DUET webserver, which contains Protein Data Bank (PDB) structures of query proteins to predict the Gibbs free energy (G) values (25).

#### RNA Expression, Gene Ontology, and Mouse Gene Knockout Model

The Human Protein Atlas (HPA) (https://www.proteinatlas.org/) database was used to determine the RNA expression status of the LD candidate gene. This database provides the expression profile of the query gene or protein based on primary antibody staining data in a series of immunohistochemistry pictures of clinical specimens. The functional enrichment analysis of the potential LD candidate gene was done using gene ontology (GO) webtool hosted in Ensembl web browser. Moreover, Mouse Genome Informatics (MGI) database (http://www.informatics.jax.org/) was used to better understand the functional role of potential LD gene on phenotype characteristics of knockout mouse models. The MGI resource provides a comprehensive set of data, tools, and analysis designed specifically for use in mouse laboratory model. It accepts input data in the form of a gene symbol and provides output corresponding to the physiological condition of knockout mice.

## Results

### Clinical Assessment

**Figure 1:**
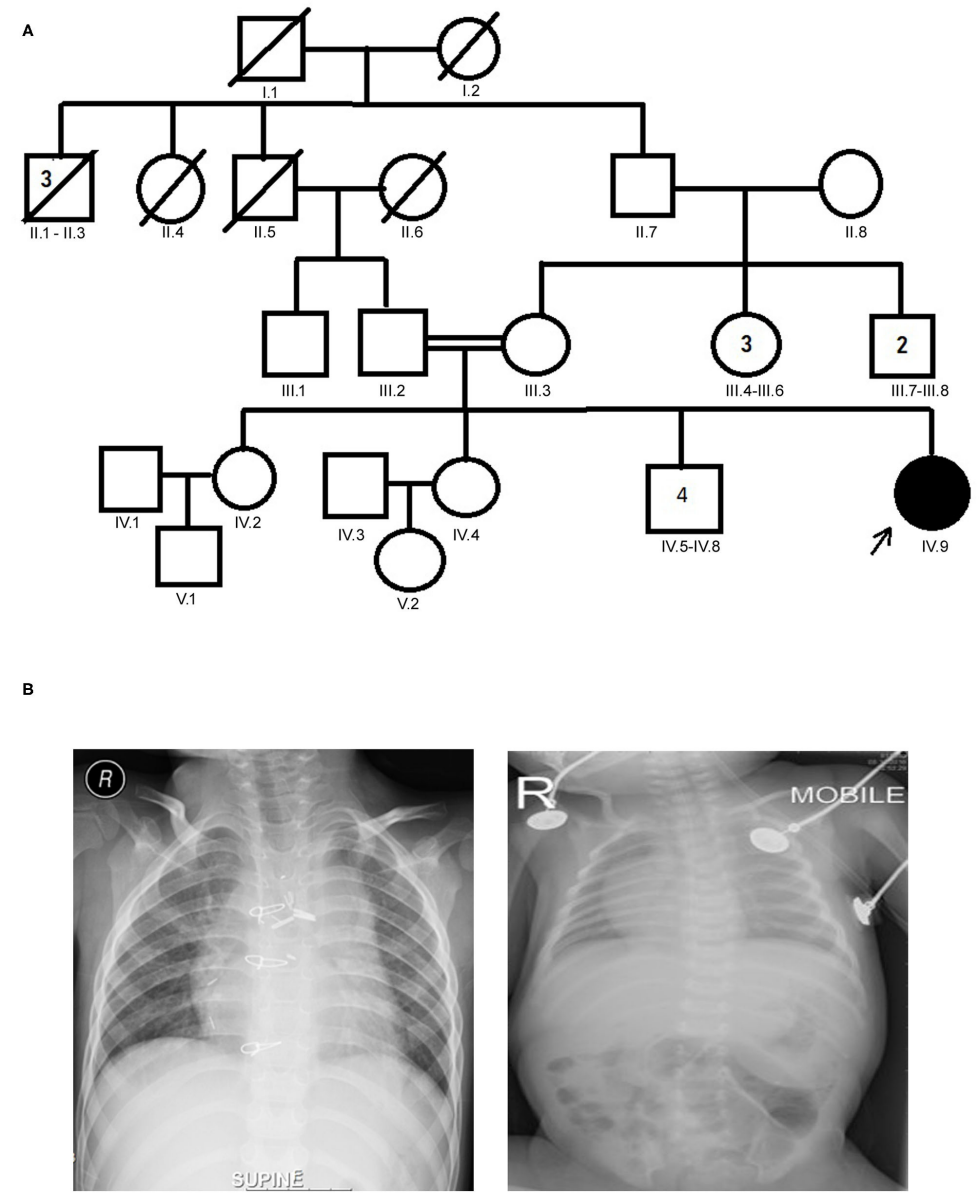
**(A)** Family pedigree chart of laterality defect (LD) Arab family. Proband is indicated by the arrow. The affected proband (shaded circle) is homozygous of c.2293C>T mutation in *MYO1D* gene. Both parents were consanguineous (double horizontal lines) and heterozygous carriers of the identified mutation. **(B)** Chest X-rays showing left isomerism (heterotaxy).

The proband aged 4 years 6 months at the time of clinical diagnosis was born to an apparently healthy consanguineous parents of Arab origin (Figure 1A). The proband exhibited a spectrum of phenotypes including visceral heterotaxy (abnormal arrangements of thoracoabdominal organs) (Figure 1B), congenital cyanotic heart disease in the form of single ventricle physiology, left isomerism with polysplenia syndrome, double inlet atrioventricular connection (a heart defect that affects the valves and chambers), pulmonary atresia, interrupted inferior vena cava with absent supra-renal segment, and azygos continuation (a rare congenital abnormality often combined with cardiovascular and visceral malformations). At the age of 10 months, the proband underwent thorough palliative cardiac procedures in the form of ductal stenting in the neonatal period followed by Kawashima cavo-pulmonary shunt (a palliative surgical procedure performed in cases of left isomerism and azygos continuation of the inferior vena cava, and common atrioventricular valve with or without regurgitation and pulmonary stenosis) in addition to left pulmonary artery (LPA) balloon dilatation procedure. At 3 years of age, Fontan completion was performed *via* incorporation of hepatic veins to pulmonary artery correcting thereby blood flow from the lower body parts directly to the lungs.

### Genetic Analysis

#### Whole-Exome Sequencing Variant Filtering and Novel Gene Identification

The sequencing of the index case generated approximately 98,000 variants, including 12,150 synonymous variants, 13,000 missense variants, and 11,500 indels. Variant filtration was based on its rare frequency, deleterious potential, autosomal recessive mode of inheritance, and functional relevance to disease (LD, primary ciliary dyskinesia (PCD), congenital heart disease, and heterotaxy). Nine genetic variants were identified as potential candidates (Table 1). Among these variants, only one missense variant (rs7209106: NM_015194.2:c.2293C>T; p.Pro765Ser) in *MYO1D* novel gene has survived our variant filtration criteria. This allele is absent in local databases like GME (Greater Middle East) (http://igm.ucsd.edu/gme/), DALIA (Disease Alleles in Arabs) (http://clingen.igib.res.in/dalia/index), and Saudi Human Genome Program (SHGP) (https://shgp.kacst.edu.sa/index.en.html#home). The MAF of this variant in international databases like 1,000 Genomes and gnomAD databases is 0.005 and 0.002, respectively. Although it has an allele frequency of 0.013 in the African population, only eight individuals are reported as homozygous for this variant in the gnomAD. But their clinical details are not provided in the gnomAD database. In the index family studied here, both parents were heterozygous and do not have any symptoms associated with LD, confirming the autosomal recessive inheritance pattern as we initially deduced from their pedigree analysis. Moreover, more than 80% (5/6; 83.34) of the computational prediction methods like CADD, FATHMM, MetaLR, Mutation Taster, PROVEAN, and REVEL have attributed pathogenicity scores to this *MYO1D* (p.Pro765Ser) variant (Table 2). Functional biology data available from model organisms like *Drosophila*, zebrafish, and frog have proved the functional role of *MYO1D* gene in LDs.

**Table 1:** List of LD potential candidate variants that showed autosomal recessive inheritance pattern.

	**Gene name**	**Chrom**	**ref**	**Alt**	**Effect**	**HGVS.c**	**HGVS.p**	**dbSNP151_ID**	**1000G_AF**	**ExAC_AF**	**gnomAD_exomes_AF**
(1)	ZC3H12A	chr1	G	T	Missense	c.99G>T	p.Arg33Ser	rs116208741	0.00139776	0.002405	0.002499959
(2)	ZC3H12A	chr1	C	T	Missense	c.95C>T	p.Pro32Leu	rs115805535	0.00119808	0.002117	0.002117
(3)	TMEM18A	chr7	C	T	Missense	c.116G>A	p.Gly39Glu	rs183593116	0.00219649	0.004772	0.00515989
(4)	CPNE7	chr16	C	T	Missense	c.898C>T	p.Pro300Ser	rs150443459	0.000399361	0.0002966	0.0003931566
(5)	ASXL2	chr2	C	T	Missense	c.1489G>A	p.Ala497Thr	rs192716734	0.00139776	0.003805	0.004480134
(6)	MYO1D	chr17	G	A	Missense	c.2293C>T	p.Pro765Ser	rs7209106	0.0043	0.002759	0.002424917
(7)	EPB42	chr15	C	T	Missense	c.1477G>A	p.Gly493Ser	rs148871144	0.000199681	0.0007578	0.0007836991
(8)	FAM220A	chr7	G	A	Missense	c.437C>T	p.Pro146Leu	rs75910050	0.00139776	0.0009884	0.0009261591
(9)	OR10A2	chr11	C	A	Missense	c.741C>A	p.Phe247Leu	rs150322658	0.00119808	0.0014	0.001421724

*LD, laterality defect*.

**Table 2:** Computational pathogenicity prediction scores of the LD candidate variants.

**S. no**	**Variant**	**Gene**	**CADD**	**FATHMM**	**MetaLR**	**MutationTaster**	**PROVEAN**	**REVEL**
(1)	rs115805535	ZC3H12A	16.06	0.22678	0.0431	0.08975	0.30964	0.027
(2)	rs116208741	ZC3H12A	–	–	–	–	–	–
(3)	rs150443459	CPNE7	–	–	–	–	–	–
(4)	rs192716734	ASXL2	14.94	0.18248	0.025	0.25126	0.24026	0.079
(5)	rs148871144	EPB42	0.135	0.78537	0.1928	0.08975	0.23156	0.121
(6)	rs75910050	FAM220A	0.524	0.06931	0.0102	0.08975	0.58248	0.049
(7)	rs150322658	OR10A2	–	–	–	–	–	–
(8)	rs183593116	TMEM184A	9.855	0.43279	0.0355	0.08975	0.07008	0.039
(9)	rs7209106	MYO1D	23.2	0.88298	0.6557	0.81001	0.80682	0.553

*LD, laterality defect*.

*CADD: >25 = damaging; <25 = tolerated; FATHMM: >0.5 = damaging; <0.5 = tolerated; MetaLR: > 0.5 = damaging; <0.5 = tolerated; Mutation Tester: >0.5 = damaging; <0.5 = tolerated; PROVEAN: >0.5 = damaging; <0.5 = tolerated; REVEL: >0.5 = damaging; <0.5 = tolerated*.

#### Sanger Sequencing Validation

**Figure 2:**
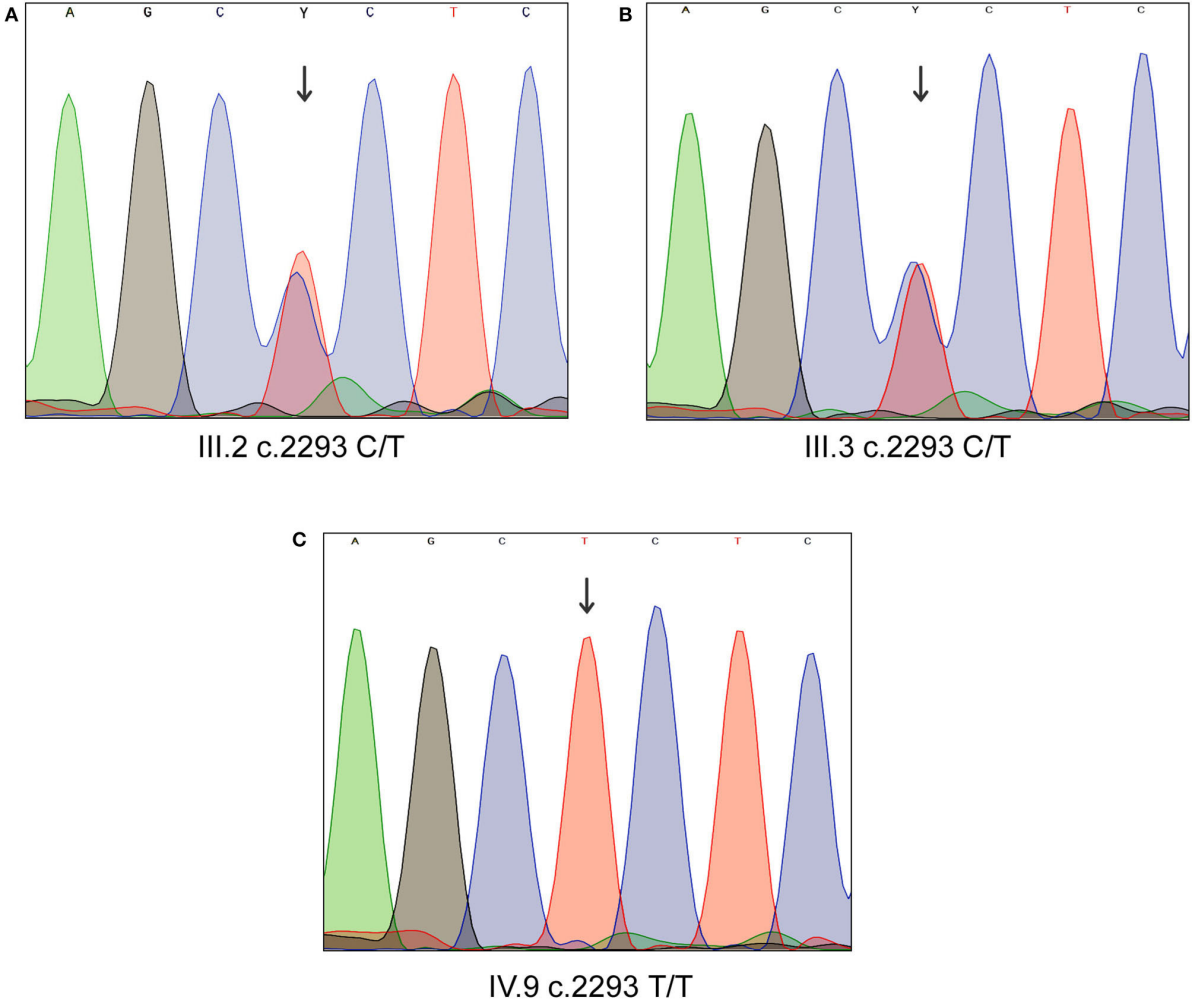
Sanger sequencing analysis of *MYO1D* gene. **(A)** Affected proband, homozygous for the variant c.2293C>T. **(B,C)** Heterozygous carriers of mother and father.

Sanger sequencing analysis confirmed that the LD patient is homozygous for c.2293C>T variant in *MYO1D* gene (1V.9, Figures 1A, 2), whereas the mother and father were heterozygous carriers (111.2, 111.3, Figures 1A, 2). This variant was absent in apparently healthy siblings and were homozygous for the T allele (1V.2, 1V.4, and 1V.5–8; Figure 1A). Two additional clinically diagnosed LD families were screened for this variant, and none carries this mutation, suggesting that *MYO1D* (p.Pro765Ser) variant is a rare private mutation in this family.

### Computational Functional Analysis

#### Variant Mapping on MYOID1 Protein Domain

**Figure 3:**
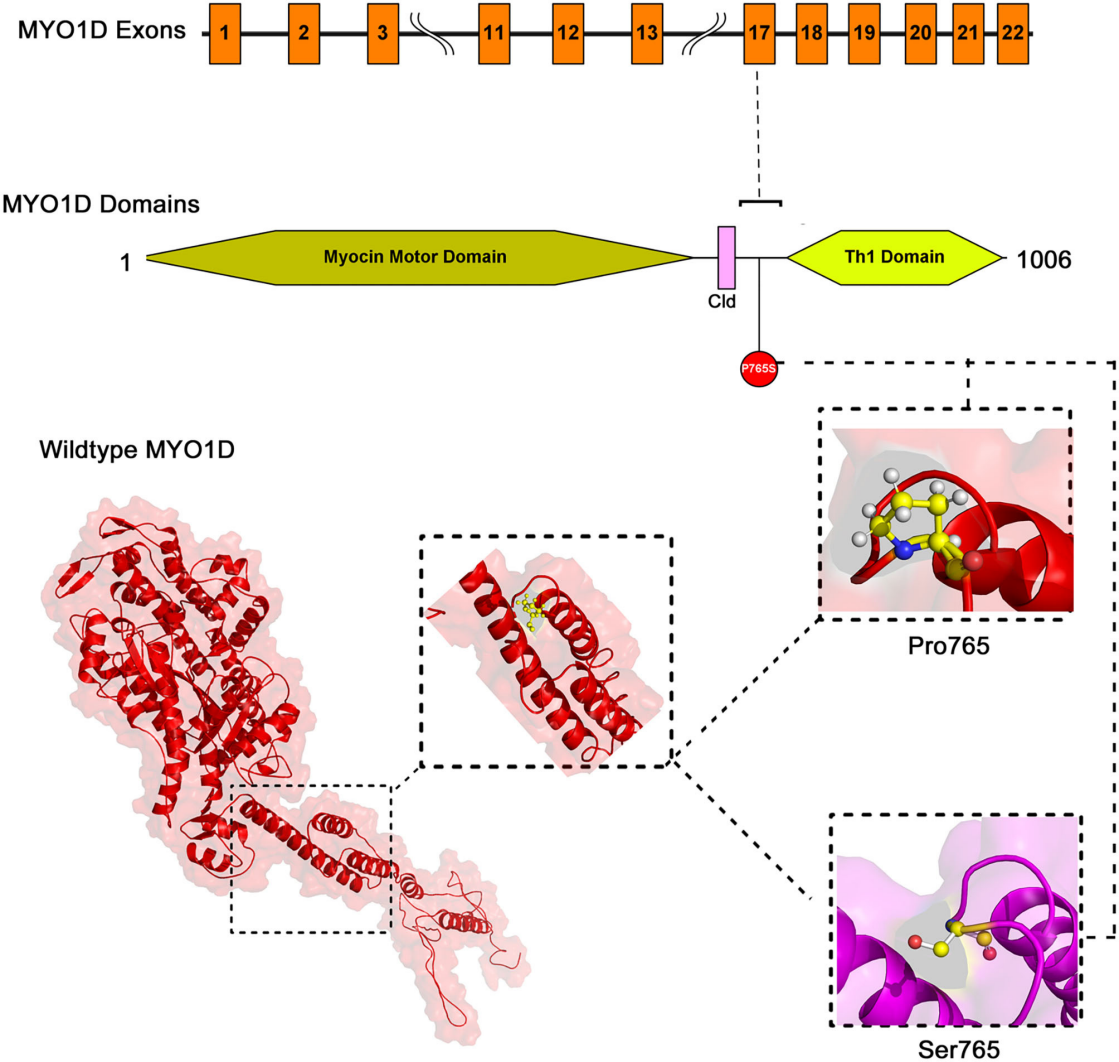
The exonic, functional domain and 3D structural annotation of *MYO1D* (Pro765Ser) variant.

The mapping of conserved amino acid domains is a vital step in deducing the association between the nucleotide sequence, protein structure, and function of disease-causing proteins. The CDD analysis showed that *MYO1D* protein is made up of three domains, namely, motor (11–682 amino acids), IQ calmodulin-binding motif (699–719 amino acids), and *Myosin TH1* (803–1,000 amino acids) domains. The Pro765Ser variant is located between the *Myosin TH1* and calmodulin-binding domains (Figure 3).

#### MYO1D 3D Model Construction

The PDB database search revealed the availability of partial 707 aa (between 10 and 717 of 1,006 AA long *MYO1D* protein) X-ray crystal protein model (4L79) with 2.3-Å resolution. Hence, the remaining 306 aa long chain was simulated with iterative threading assembly modification (I-TASSER) webserver following an *ab initio* approach. From the I-TASSER output, the best *MYO1D* model was chosen based on its polypeptide prediction quality scores like confidence score (C = −1.52), template modelling (TM = 0.53 ± 0.15), and root-mean-square deviation (RMSD) (12.7 ± 4.3 Å) scores. These quality metrics indicate the very good structural similarity between the query and template proteins (Figure 3). The stereochemical evaluation of the energy-minimised *MYO1D* protein model revealed that 96.2% of the amino acids are in the allowed portion of the protein, whereas 3.8% are in the non-allowed region. As per the above outlined processes, the native MYO1D model was used as a template to create a mutant variant by manually substituting proline for serine at the 765th position. PyMOL was used to depict native and mutant proteins.

#### Structural Deviation and Stability Findings

We have used YASARA tool to analyze Cα-atom coordinates of native and mutant *MYO1D* 3D structures to evaluate their structural drifts (in terms of RMSD) at residue and whole structure levels. RMSD value is used to quantitatively measure the structural similarity between two atomic coordinates when superimposed on each other. The impact of substitution mutations on amino acid structures can be calculated when there is a divergence at the polypeptide chain level. We noticed minor structural drifts in *MYO1D* structure only at 765th residue position due to the RMSD value difference (2.28) induced by the substitution of proline with serine (Figure 3). The DUET analysis of the *MYO1D* (P765S) variant predicted Gibbs free energy (ΔΔG) alterations shifting the energy equilibrium to negative value, i.e., −0.959 kcal/mol, suggesting that the queried variant is potentially deleterious to the protein stability owing to its destabilising behaviour.

#### RNA Expression Analysis

**Figure 4:**
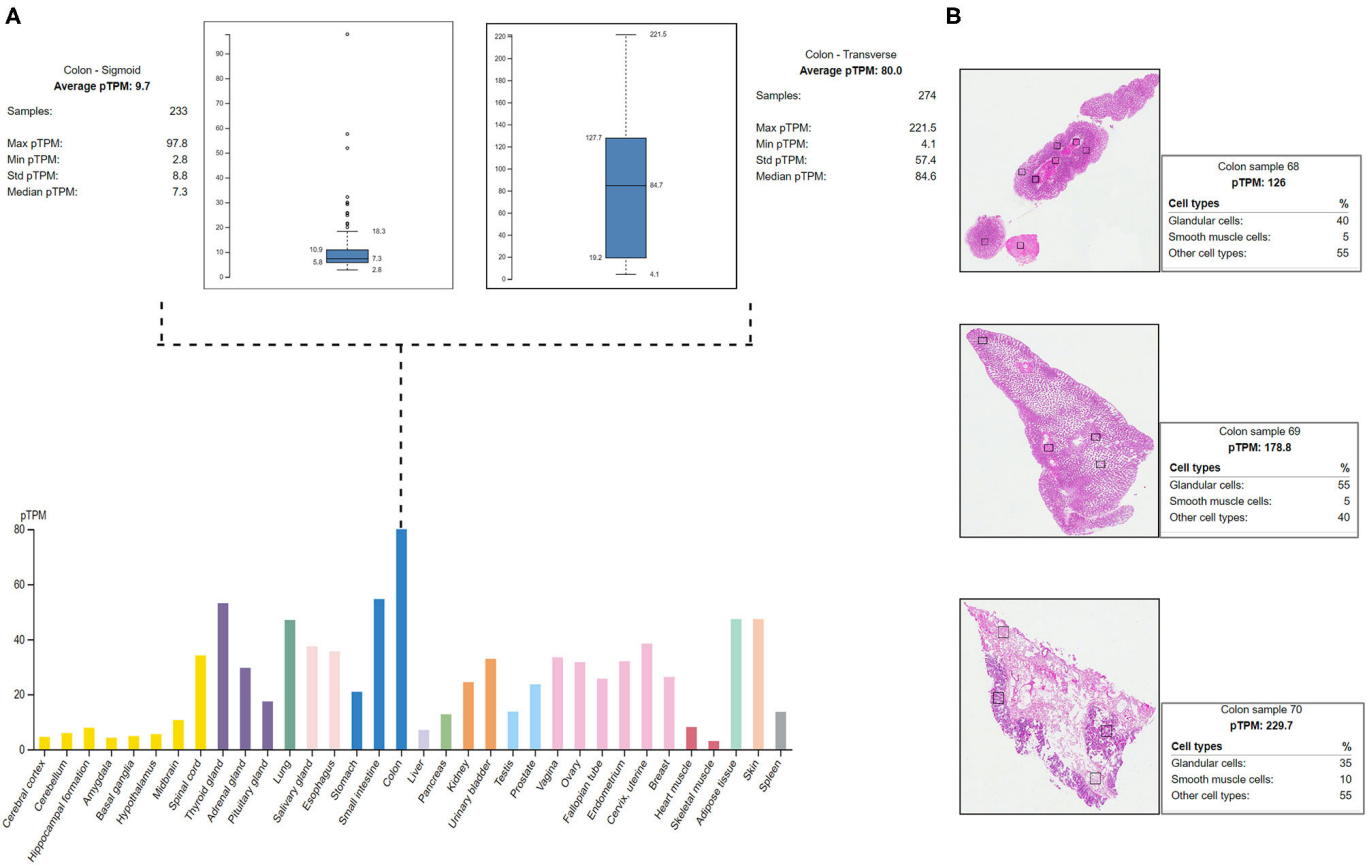
Protein Atlas expression analysis of *MYO1D*. **(A)** Bar graph represents the *MYO1D* expression in sigmoid and transverse colon samples. **(B)** Histopathological examinations of colonic samples from three different patients showing the *MYO1D* protein expression.

The HPA shows the positive expression status of *MYO1D* gene in different tissues and organs of the human body like the colon, lungs, and thyroid gland. In particular, the highest expression was seen in the digestive system with the colon, where the 274 transverse colon samples showed a maximum of 221.5 protein transcripts per million (pTPM) and 233 sigmoid colon samples showed a maximum of 97.8 pTPM. The RNA-Seq analysis of immunohistochemistry tissue specimens from three control specimens showed that glandular cells showed the highest pTPM status of *MYO1D* gene when compared with smooth muscle cells and other cell types in the colon (Figure 4).

#### Gene Ontology and Gene Knockout Analysis

The Ensembl GO analysis of *MYO1D* gene showed its involvement in 43 GO terms, including those connected to biological processes (seven GO terms), molecular functions (11 GO terms), and cellular component (25 GO terms) (Supplementary Table 1). All the annotations collectively highlight that *MYO1D* is localised in the cytoplasm (cellular component) and plays an important role in actin filament organisation (biological process) as well as microfilament motor activity function (molecular function). Supplementary Table 2 shows details of disease phenotypes corresponding to the *MYO1D* genetic background in different knockout mice models.

## Discussion

Recent evolution and easy accessibility of next-generation sequencing resulted in accurate molecular diagnosis of variety of genetic diseases from around the globe (39, 40). Owing to the genetic heterogeneity, identification of specific molecular cause of LD is very challenging in up to 80% of the cases. Majority of known LD causative genes are structural proteins of the cilia and are known for their involvement in NODAL/TGFβ or SHH signalling pathways. During embryonic development, these genes play an important role in symmetrical LR positioning of the organs (10).

*MYO1D* gene consists of 27 exons mapped to chromosome 17q11.2. Myosin heavy chain class 1 is a member of the myosin superfamily, playing essential roles in cytoskeletal structure, mechanical signal-transduction membrane dynamics (41), and endosome processing (42). In the present study, we identified the first case of a homozygous missense (c.2293C>T) mutation in *MYO1D* gene causing LD in the proband of an Arab consanguineous family. Further screening of LD participants from two additional Arab families did not reveal any mutations in this gene. In Middle Eastern Arab databases (with more than 10,000 exome data combined) such as SHGP, GMC, and DALIA, not a single case was recorded for this variant. Also, this variant was extremely rare (<0.005) in 1,000 Genomes and 0.013 in gnomAD across all ethnic groups. In gnomAD, only eight individuals were reported as homozygous (six males and two females), but no clinical data were available.

Various studies suggested the role of *MYO1D* gene in laterality disease in *Drosophila*, zebrafish, and frog (43–47). *MYO1D* has a role in organ asymmetry in *Drosophila*, which lacks cilia and nodal pathway while developing LD by using polar cell polarity (PCP), *MYO1D*, and *HOX* gene Abd-B (48, 49). Another study demonstrated the function of *MYO1D* in *Xenopus laevis* and influence the orientation of the cilia on the LR organiser (LRO) through planar cell polarity pathway as implicated in *Drosophila* (45). In zebrafish, *MYO1D* plays a fundamental role in the LR organisation (43, 50). Aside from the above initial reports, our understanding of *MYO1D* function in the context of human LR patterning remains largely unexplored. The *situs inversus* (SI) phenotype is reported in approximately 50% of PCD cases with congenital cardiac defects (51, 52). Approximately 3–7% of LD patients have CHDs (53). Many studies (54, 55) in a variety of species failed to identify a unifying mechanism for LR patterning. However, recent studies (43, 50) provided the first evidence of a shared origin of laterality in both arthropods and chordates through *MYO1D* gene. The clinical features are consistent with previous observations in zebrafish and *Drosophila*, indicating that MYO1D has an important role in LR patterning during embryogenesis. Moreover, asymmetric clustering of cilia was disrupted in ependymal cells of *MYO1D* KO rat models, consistent with LD (56).

**Figure 5:**
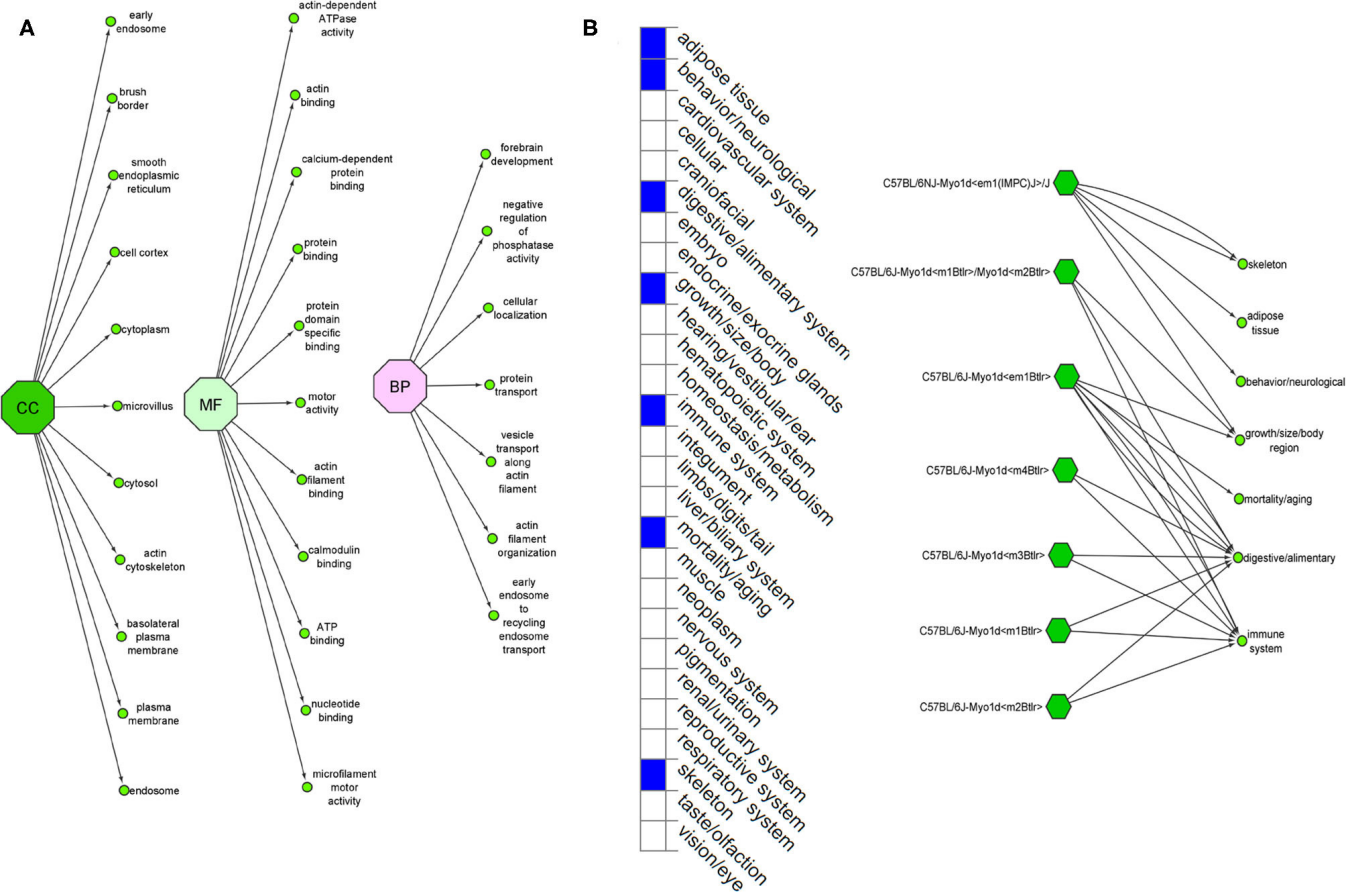
**(A)** Ensembl function annotations of *MYO1D*. **(B)** Mouse *MYO1D* knockout analysis and phenotypic changes.

*MYO1D* gene knockout in different mouse strains (seven different strains) is shown to demonstrate a variety of phenotypes like decreased body fat amount (adipose tissue), decreased startle reflex (behaviour/neurological), increased susceptibility to colitis (digestive/alimentary), decreased bone mineral content (skeleton), and increased susceptibility to weight loss (growth size/body weight and immune system and mortality or ageing) (Figure 5). Our study is the first one to report the association of defective *MYO1D* to LD in humans, confirming that the function is evolutionarily conserved from *Drosophila*, to zebrafish, to frog, to humans. Thus, *MYO1D* gene can be considered as the new causal gene for LD in humans. Though the *Drosophila* and zebrafish models clearly showed the visceral heterotaxy, mouse KO phenotypes were surprisingly not showing any LD-related phenotypes.

Extensive computational analysis of the protein structure and function adds the supporting evidence for *MYO1D* in LD. *MYO1D* protein is 1,006 aa long with a molecular weight of 116 kDa. It consists of a large, highly conserved Myocin Motor Domain (671 aa), short calmodulin-binding motif (20 aa), and a basic C-terminal tail homology-1 (TH1) domain (197 aa). The amino acid residue level structural deviation observed with the variant serine (Pro765Ser) in *MYO1D* is likely to disturb the primary, secondary, tertiary, and quaternary structural features in the protein. Numerous studies have shown the strong correlation between deviations in residue level RMSD score and structural properties for the disease-causing variants (23, 57–59). Disease causative pathogenic mutations have often changed the energy equilibrium, which is required to maintain the protein stability (60). Given the close physical proximity of P765S variant between calmodulin-binding motif and TH1 domains, the conformational and stability changes in *MYO1D* protein are likely to impact its main biological functions such as calmodulin binding, actin-dependent ATPase activity, calcium-dependent protein binding, and microfilament motor activities (61).

LD is a complex disease, and its clinical phenotype presentations often overlap with PCD symptoms. A recent study from Saudi Arabia reported the overlapping clinical symptoms between PCD and LD patients (26). This report investigated a total of 81 patients, including 58 patients with sinopulmonary infections (SPIs), 15 patients with combined LD with SPIs, and six patients with LD alone. They reported mutations in the known PCD genes as follows: *RSPH9, CCNO*, DNAAF5, *RSPH4A, MCIDAS*, and *CCDC40* gene mutations in PCD patients with SPIs; *CCDC151, DNAH11, CCDC40, DNAH5*, and *CCDC39* gene mutations in LD patients with SPIs; *PKD1L1* and *DNAAF5* gene mutations in LD patients; and *RSPH9* and *MCIDAS* gene mutations in neonatal respiratory distress. Additionally, they have also identified gene mutations in *ITCH* and *CEP164* in two patients, demonstrating ITCH-related syndrome and Bardet–Biedl syndrome. Sparrow et al. (29) reported *HES7* as a cause of spondylocostal dysostosis with SI and dextrocardia. Molecular diagnosis of LD and PCD in Arab patients has revealed a spectrum of mutations in many genes with variable clinical presentations (35), which is summarised in Table 3.

**Table 3:** Phenotypes and genetic data of LD and/or PCD among Arabs.

**Nationality**	**Situs inversus (SI)**	**PCD**	**Dextrocardia**	**Heterotaxy of abdominal organs**	**Recurrent chest infections**	**Chronic rhinitis**	**Bronchiectasis**	**Chronic or recurrent otitis media**	**Gene**	**Reference**
Yemeni	+	–	+	Visceral heterotaxy, polysplenia syndrome	–	–	–	–	*MYO1D*	Present study
Saudi (6 patients)	+6/6	–	NR	NR	–	NR	NR	NR	*DNAH5, PKD1L1, DNAAF5*/*CYP21A2, DNAI1*	(26)
Arab	+	NR	+	NR	NR	NR	NR	NR	*GDF1*	(27)
Saudi	+	NR	+	Abdominal ultrasound revealed that the liver and gallbladder were located in the left hypochondrium, spleen on the right side	NR	NR	NR	NR	*NR*	(28)
Arab	+	NR	+	+	NR	NR	NR	NR	*HES7*	(29)
Saudi (75 patients)	+	+73/75	NR	NR	+15/75	NR	NR	NR	*CCDC151, CCDC39, CCDC40, DNAH11, GOLGA3, RSPH9, CCNO, RSPH9, ITCH, MCIDAS, RSPH4A, DNAH5, CEP164, GOLGA3*	(26)
Morocco	+	+	+	+	+	+	+	–	*NR*	(30)
Saudi	+	+	+	NR	NR	+	+	+	*DNAH1*	(31)
Kuwaiti	+	+		NR	NR	+	NR	NR	*DNAH5*	(32),
Saudi	NR	+	NR	NR	+	+	+	NR	*CCDC151*	(33)
Palestine	NR	+	+	NR	NR	NR	+	+	*LRRC6*	(34)
Saudi	NR	+	NR	NR	NR	NR	+	NR	*RSPH9*	(35)
UAE	NR	+	NR	NR	+	NR	+	NR	*RSPH9*	(36)
UAE	–	+	NR	NR	+	+	+	+	*RSPH9*	(37)
Saudi	+	+	NR	NR	NR	NR	+	NR	*19q13.3*	(27)
Saudi	+	+	–	Situs inversus of cardiac shadow and gastric air bubble	+	NR	+	–	*NR*	(38)

*+, presence of symptoms; –, absence of symptoms; NR, not reported; PCD, primary ciliary dyskinesia*.

## Conclusion

In conclusion, we discovered missense mutation in *MYO1D* gene (c.2293C>T) in an Arab patient presenting with visceral heterotaxy and left isomerism with polysplenia syndrome by using higher-throughput WES technology. This is the first report to establish the relationship between *MYO1D* variants and LD, supporting the previous findings in *Drosophila zebrafish*, and frog. This exciting finding may support the critical role of *MYO1D* gene for LR patterning in humans. This study has some sincere limitations, as this is the first case identified with *MYO1D* mutation potentially contributing to LD phenotypes, and there are no reported cases with *MYO1D* variants to compare our data with. Therefore, testing *MYO1D* variants for LD patients in large cohort studies is recommended to verify our findings. Future functional studies are also recommended to investigate the specific molecular role and therapeutic prospects of targeting *MYO1D* genetic variants in patients demonstrating LD phenotypes.

## Data Availability Statement

The datasets presented in this article are not readily available because (a) participants' refusal to store or distribute the genomic data in the public domain and (b) as per the local Institutional Ethics committee approval and Saudi national policy on genomic data sharing in the public domain outside the country. Requests to access the data should be directed to RE or TS.

## Ethics Statement

The Ethical approval for the present study was obtained from institutional Ethics Committee of King Abdulaziz University Hospital (KAUH), Jeddah, Saudi Arabia and informed consent forms were collected from both adult parents and their children (guardian consent for Language evaluation).

## Consent to Participate

Informed consent was obtained from all subjects involved in the study.

## Author Contributions

TA, RE, and KN: conceptualisation. KN, RA, BB, HE, and RE: methodology. BB: software and visualisation. KN, RA, BB, NS, and RE: formal analysis. RA, KN, TS, and RE: investigation. KN, TS, and BB: resources. RA, KN, TS, BB, ZZ, GA, NS, JA-A, AS, OA-R, RE, and TA: writing—original draft preparation. KN, TS, NS, and RE: writing—review and editing. KN, BB, and RE: supervision. TS: project administration and funding acquisition. All authors contributed to the article and approved the submitted version.

## Funding

This research work was funded by Institutional Fund Projects under grant no. (IFPRC-132-290-2020). Therefore, authors gratefully acknowledge technical and financial support from the Ministry of Education and King Abdulaziz University, Jeddah, Saudi Arabia.

## Conflict of Interest

The authors declare that the research was conducted in the absence of any commercial or financial relationships that could be construed as a potential conflict of interest.

## Publisher's Note

All claims expressed in this article are solely those of the authors and do not necessarily represent those of their affiliated organizations, or those of the publisher, the editors and the reviewers. Any product that may be evaluated in this article, or claim that may be made by its manufacturer, is not guaranteed or endorsed by the publisher.
